# Parkinson's disease as a result of aging

**DOI:** 10.1111/acel.12312

**Published:** 2015-02-09

**Authors:** Manuel Rodriguez, Clara Rodriguez-Sabate, Ingrid Morales, Alberto Sanchez, Magdalena Sabate

**Affiliations:** 1 Laboratory of Neurobiology and Experimental Neurology, Department of Physiology, Faculty of Medicine, University of La LagunaLa Laguna, Spain; 2 Center for Networked Biomedical Research in Neurodegenerative Diseases (CIBERNED)La Laguna, Spain; 3 Rehabilitation Service, Department of Pharmacology and Physical Medicine, Faculty of Medicine, University of La LagunaLa Laguna, Spain

**Keywords:** aging, dopamine, nigrostriatal neurons, neurodegeneration, Parkinson's disease

## Abstract

It is generally considered that Parkinson's disease is induced by specific agents that degenerate a clearly defined population of dopaminergic neurons. Data commented in this review suggest that this assumption is not as clear as is often thought and that aging may be critical for Parkinson's disease. Neurons degenerating in Parkinson's disease also degenerate in normal aging, and the different agents involved in the etiology of this illness are also involved in aging. Senescence is a wider phenomenon affecting cells all over the body, whereas Parkinson's disease seems to be restricted to certain brain centers and cell populations. However, reviewed data suggest that Parkinson's disease may be a local expression of aging on cell populations which, by their characteristics (high number of synaptic terminals and mitochondria, unmyelinated axons, etc.), are highly vulnerable to the agents promoting aging. The development of new knowledge about Parkinson's disease could be accelerated if the research on aging and Parkinson's disease were planned together, and the perspective provided by gerontology gains relevance in this field.

Aging is characterized by a progressive decline of many physiological functions, an increased susceptibility to certain diseases, and an increase in the likelihood of death. It is a complex phenomenon that may be very diverse in the different animal species, in individuals of the same species, and in the different tissues of the same individual (Miller, 1999; Takubo *et al*., 2002; de Magalhaes & Costa, 2009). Although there are a number of different theories to explain the onset and progression of aging, it is generally accepted that aging is not the result of one single cause. The causal agents and the mechanisms that collaborate to produce aging can be divided into two groups: passive agents inducing tissue damage which are not properly repaired and which accumulate throughout life (*damage-based theories*), and genetic agents that actively alter the physiological behavior of cells (*programmed theories*). Both theory types generally assume that aging is a multifactor phenomenon where the equilibrium between cell damage and cell repair is progressively lost throughout life (*multifactor hypothesis*). This imbalance is not evoked in the brain in the same way in the different cell types, with the glial and the neuronal subgroups comprising each center showing a different aging rate (Going *et al*., 2002; Jeyapalan & Sedivy, 2008). This study revises the information available about a subgroup of neurons [dopamine neuron (DAn)] of a mesencephalic center [substantia nigra (SN)] which is involved in a neurodegenerative illness [Parkinson's disease (PD)]. PD is generally considered as a single clinical entity with a specific etiopathogenesis. The possibility that PD could be the consequence of the degeneration of neurons which are particularly vulnerable to aging processes is discussed here.

## PD and aging

PD is a slow-progression neurodegenerative disorder with a high incidence in aged people. Aging is the greatest risk factor for PD (Driver *et al*., 2009; Collier *et al*., 2011), and the incidence of the illness in people over the age of sixty increases in an exponential way (Driver *et al*., 2009). Tables of the trends in the health of the older population in the United States show that PD is present in 0.02% of people who died between 45 and 54 years of age and in 8.77% of people who died over 85 years of age. The prevalence of other diseases (e.g. heart disease or Alzheimer's disease) also increases with aging, but none of them show the marked increase found in PD whose prevalence increases more than 400 times with aging. In addition, aging has been considered as the most important determinant of the clinical worsening of patients with PD, even when compared with variables such as disease duration (Levy, 2007; Collier *et al*., 2011). The response to treatments (e.g. levodopa) is also hampered by the advanced age (Levy, 2007). Thus, aging is a variable which is always present in PD.

The marked influence of aging on PD could explain the scarce information referring to the illness before the 19th century (there are a few documents from ancient India and Greece and from the middle ages in Europe referring to parkinsonian signs with names such as kampavata, paradoxos, and shaking palsy). The initial mortality rate (mortality rate independent of aging) was high before the 19th century, and most people did not reach the age where the incidence of PD becomes relevant. Life expectancy increased in such a way during the 19th century as to attract the attention of James Parkinson (1817). This increase was spectacular during the 20th century, making PD a very important health problem which in the USA alone affects 59 000 new people every year (Hirtz *et al*., 2007; Driver *et al*., 2009; Wirdefeldt *et al*., 2011).

## Degeneration of dopamine cells in PD and aging

Neurodegeneration in PD occurs in different brain centers that also degenerate with age (locus coeruleus, pedunculopontine nucleus, etc.; Lohr & Jeste, 1988; Ransmayr *et al*., 2000; Shibata *et al*., 2006). The mechanisms involved in the degeneration of these centers are not very well known, which limits the possibility of comparing the PD and aging neurodegeneration in these centers. This is not the case of SN cells whose degeneration is currently considered as the hallmark of PD and whose change with aging has been extensively documented. Thus, most comments in this review will be focused on the neuronal degeneration in the SN.

The initial studies showing neurodegeneration in the SN of patients with PD also reported a marked neuronal loss in the SN of the healthy subjects included in the control groups of these studies (Hirai, 1968; McGeer *et al*., 1977; Stark & Pakkenberg, 2004; Mortera & Herculano-Houzel, 2012). These studies were focused on the SN because this center loses its characteristic dark gray color in the PD brain, which is normally produced by the accumulation of the pigment neuromelanin in a subgroup of SN neurons. PD showed a decrease in nigral pigmented neurons (Hirsch *et al*., 1988, 1989; Fearnley & Lees, 1991), which suggested that the selective degeneration of these cells could be a feature of this illness and that neuromelanin may be involved in this degeneration (Hirsch *et al*., 1988). However, healthy aged subjects also showed a decrease in pigmented cells (7-10% decrease per decade) (McGeer *et al*., 1988; Ma *et al*., 1999; Cabello *et al*., 2002; Stark & Pakkenberg, 2004; Fedorow *et al*., 2005; Halliday *et al*., 2006; Double *et al*., 2008; Rudow *et al*., 2008), suggesting that if neuromelanin was involved in PD, it could also be involved in aging-related neurodegeneration.

The neuromelanin accumulation in the SN neuron (SNn) is the result of dopamine (DA) oxidation, which, together with the therapeutic effects of levodopa therapy in PD and the DA decrease found in the striatum of these patients (Hornykiewicz, 2008, 2010), suggested the DAn degeneration as the basis of PD. The neuromelanin+ neuron count is not the best way to estimate the number of DAn because the pigment slowly accumulates throughout life (which hampers the identification of DAn in young subjects) and because a portion of DAn can suffer a de-differentiation in PD (which means that the number of neuromelanin+ cells can be higher than those expressing a dopaminergic phenotype; Gonzalez-Hernandez *et al*., 2004; Kordower *et al*., 2013). Immunohistochemical markers to detect proteins involved in the synthesis (e.g. tyrosine hydroxylase and l-dopa decarboxylase), degradation (monoamine oxidase), and membrane transport (dopamine transporter (DAT)) of DA proved to be a better procedure to estimate the number of DA neurons. Different studies in patients with PD reported neurodegeneration of SN cells expressing DAT (McGeer *et al*., 1977; Kastner *et al*., 1993; Miller *et al*., 1999; Chu *et al*., 2006; Rudow *et al*., 2008; Kordower *et al*., 2013; Ishibashi *et al*., 2014) and the enzymatic machinery necessary for the degradation of DA (Lloyd & Hornykiewicz, 1970; Grote *et al*., 1974; Robinson *et al*., 1977; Saura *et al*., 1997). This cell loss is often considered as being selective for PD and is generally used as the keystone to diagnose the illness with positron emission tomography and other methods. However, most studies also found a DAn loss in the SN of healthy aged subjects, with the quantity of the cell loss being the main difference between PD and aging. The nigral distribution of the DAn degeneration has also been proposed as being selective for PD, with the ventral tier of the posterior and lateral regions of the SN compacta showing the highest degeneration rate (Fearnley & Lees, 1991; Damier *et al*., 1999; Rodriguez Diaz *et al*., 2001; Rodriguez *et al*., 2001). However, this DAn subgroup has also shown the highest rate of degeneration in aged monkeys (Kanaan *et al*., 2008; Collier *et al*., 2011) and healthy aged humans (Reeve *et al*., 2014), suggesting that the distribution of DAn degeneration is not selective for PD either.

On the other hand, the DAn degeneration during aging is a common phenomenon observed in animals and humans (Collier *et al*., 2011). A recent work on 750 aged brains of subjects which never showed a clinically defined PD reported a marked DAn loss in more than 30% of cases, which in addition showed other pathological characteristics of PD such as the Lewy bodies (Buchman *et al*., 2012). Most nigral DA neurons project to the striatum, forming the nigrostriatal DAn (snDAn). system. It has been found that the slow degeneration of these cells with aging may last for years without inducing clinical signs of PD, signs which cannot be found when the snDAn degeneration does not involve more than 50% of these cells or more than 70% of their synaptic terminals in the striatum. Thus, the difference between PD and aging brains could be a question of quantity (number of DAn lost) more than a question of quality (the type of cell which degenerates) (McGeer *et al*., 1988).

## Physiological changes of DAn in PD and aging

DA neurons may experience physiological changes during aging that are similar to those observed in these cells before their degeneration in PD. The striatal decrease of DA observed in PD has also been found in the aged brain where the DA level decreases 10–13% per decade of life (Carlsson & Winblad, 1976; Riederer & Wuketich, 1976; Kish *et al*., 1992). The topographic distribution of the striatal DAergic denervation observed in PD (Kish *et al*., 1988; Hornykiewicz, 1989) has also been reported in the aged brain (Kish *et al*., 1992; Haycock *et al*., 2003). There are indications that an increase in DA turnover might be an early compensatory mechanism for DAn degeneration in PD (Barrio *et al*., 1990; Rodriguez & Castro, 1991; Sossi *et al*., 2002). A similar compensatory mechanism has been proposed for the age-related loss of DAn (Greenwood *et al*., 1991). There is evidence suggesting that nsDAn surviving the SN degeneration may present a partial loss of their phenotypic characteristics in the PD (Kastner *et al*., 1993; Miller *et al*., 1999; Gonzalez-Hernandez *et al*., 2001, 2004; Chu *et al*., 2006), a fact that could explain why a portion of the melanin-positive SN neurons does not express TH or other dopaminergic markers in PD (Kordower *et al*., 2013). DAn degeneration probably begins in the distal axon and proceeds retrogradely (dying-back process) (Galvin *et al*., 1999; Cheng *et al*., 2010), inducing an early inhibition of the fast axonal transport (Coleman, 2005; Morfini *et al*., 2007; De Vos *et al*., 2008; Neukomm & Freeman, 2014) accompanied by a decreased expression of the dopaminergic phenotype (Bellinger *et al*., 2011; Cheng *et al*., 2011). A similar decrease in the dopaminergic phenotype has been found in the snDAn which survive to a partial degeneration of the nigrostriatal system (which may change its DAergic phenotype by a GABAergic phenotype) (Rodriguez & Gonzalez-Hernandez, 1999; Gonzalez-Hernandez *et al*., 2001), and in nigrostriatal cells of animals with a degeneration of the contralateral snDAn (Gonzalez-Hernandez *et al*., 2004). The phenotype of neurons may be also modified by aging (Tedroff *et al*., 1988; Volkow *et al*., 1996; Emborg *et al*., 1998; Mozley *et al*., 1999; Pirker *et al*., 2000; Gerhardt *et al*., 2002; Kanaan *et al*., 2007), a fact observed in SN neurons which gradually loss their DAergic phenotype with aging whereas preserve the cytoplasmic neuromelanin (McCormack *et al*., 2004). D1 and D2 dopamine receptors present a compensatory increase in the brain of untreated PD (Lee *et al*., 1978; Centonze *et al*., 2003), an event also found in the aged brain (Grote *et al*., 1974; Morgan *et al*., 1987; Suhara *et al*., 1991; Wang *et al*., 1998b) (although some inconsistent data have also been reported) (Hess *et al*., 1987; Suhara *et al*., 1991; Wang *et al*., 1998b). Thus, functional changes observed in the nsDAn surviving the PD neurodegeneration have also been found during healthy aging. Most of these changes are probably an attempt by the surviving neurons to compensate for the partial degeneration of the other DA neurons (increase of DA turnover and DA receptors) or to prevent their own degeneration (loss of phenotypic characteristics), thereby suggesting that the physiological response to the partial loss of the nsDAn system could be similar in the PD and aged brain.

## Degeneration of nondopamine neurons in PD and aging

As mentioned above, the snDAn is not the only neuron which degenerates in PD. DA neurons of the ventral tegmental area and retrorubral field (..), and other neurons interconnected with these cells such as the noradrenergic neurons of the locus coeruleus (?), cholinergic neurons of the pedunculopontine nucleus (?), and glutamatergic neurons of the intralaminar thalamic nucleus (?), also degenerate in PD. In addition, PD neurodegeneration has been found in neurons not directly interacting with the snDAn, such as those of the myenteric plexus or the olfactory bulb. The mechanisms involved in the loss of non-DA neurons have been little studied, and there is evidence supporting the idea that PD degeneration begins in non-DA neurons and then progresses to DA neurons (Braak *et al*., 2003; Ferrer *et al*., 2011), but also supporting the possibility that the degeneration of non-DA neurons could be induced by the previous degeneration of DA neurons (Morales *et al*., 2013a,b). In any case, the parkinsonian degeneration observed in most non-DA neurons has also been found in the healthy aged brain (Iversen *et al*., 1983; Baker *et al*., 1989; Manaye *et al*., 1995; Arango *et al*., 1996; Ransmayr *et al*., 2000), which also presents the cytosolic aggregates reported in the PD brain (e.g. Lewy bodies) (Mann, 1983; Halliday, 2009). The systematic study of cases with protein aggregate pathology has prompted a staging classification of PD based on the putative progression of Lewy body pathology from the medulla oblongata and olfactory bulb to the midbrain, diencephalon, and neocortex (Braak *et al*., 2002, 2003; Muller *et al*., 2005; Braak *et al*., 2006). In the aforementioned proposals, the DAn degeneration is not substantial (<60% of cell loss) before stage 3 is reached, with stages 1 and 2 being characterized by the presence of aggregates outside the SN (e.g. myenteric plexus, dorsal IX/X motor nuclei, intermediate reticular zone, caudal raphe nuclei, locus coeruleus, and olfactory bulb) and by the existence of nonmotor clinical disturbances (Jellinger, 2002; Ferrer *et al*., 2011). Much research is being carried out to identify early nonmotor signs which can be used to diagnose PD during its premotor stages. This trial is hampered by the fact that most premotor clinical signs of PD (e.g. weight loss, olfactory dysfunctions, sleep fragmentation, constipation, and mood disorders) are also found in healthy aged subjects (Pfeiffer, 2003, 2010; Cersosimo & Benarroch, 2011; Doty, 2012). Thus, neither the degeneration of non-DA cells nor the presentation of nonmotor disturbances associated with it can clearly distinguish PD and aging (Double *et al*., 2010), suggesting that the degeneration of these cells may not be substantially different in aging and PD.

## The involvement of non-neuronal cells in PD and aging

The anomalous activity of glial cells has been involved in the progression of PD. Although the glial dysfunctions of PD are frequently considered as being specific to the illness, most of them have also been observed in the aged brain. Both aged and PD brains present a low-level chronic inflammation (‘neuro-inflammaging') with complex changes in the activity of astrocytes and microglia (Franceschi *et al*., 2007; Chung *et al*., 2009). Both cells may produce detrimental effects on neighboring neurons due to chronic production of pro-inflammatory agents (ROS, leukocyte-attracting cytokines, etc.). However, both cells may also provide structural, metabolic, and trophic support to neurons (Allen & Barres, 2009; Chung *et al*., 2009; Nagelhus *et al*., 2013). This ambivalent activity is hampering the study of the role of the glial cells in PD.

Astrocytes are involved in a variety of physiological functions (Benarroch, 2009; Sofroniew & Vinters, 2010; Rodriguez *et al*., 2012) and whose deterioration has been linked to both PD and aging (Raivich *et al*., 1999; Sofroniew & Vinters, 2010; Belanger *et al*., 2011; Rodriguez *et al*., 2012; Morales *et al*., 2013a). These cells may behave in an opposing manner, promoting damage or providing protection to neurons. Astrocytes synthesize glutathione (Rice & Russo-Menna, 1998) whose release protects neighboring cells from oxidation (Hirrlinger *et al*., 2002), a protection which is lower in PD (Zeevalk *et al*., 2008). Astrocytes produce trophic factors (basic fibroblast growth factors, mesencephalic astrocyte-derived neurotrophic factor, glial cell line-derived neurotrophic factor (GDNF), etc.) that protect the DAn from damage (Lin *et al*., 1993; Saavedra *et al*., 2006; Deierborg *et al*., 2008). In addition, astrocytes remove toxic molecules from the extracellular medium, including the α-synuclein that has escaped from axon terminals (which prevents its aggregation in DAn) (Braak *et al*., 2007; Song *et al*., 2009; Lee *et al*., 2010) and glutamate released from glutamatergic neurons (which prevents its excitotoxic action) (Rodriguez *et al*., 2012; Morales *et al*., 2013a,b). These protective activities decrease during aging. Astrocytes cultured from the brain of aging rats show morphologic and staining (e.g. beta-galactosidase.) patterns which are characteristic of senescence, reducing their ability to protect neurons (Mansour *et al*., 2008; Chinta *et al*., 2013). These senescent astrocytes are involved in the neuronal loss in the aged brain (Pertusa *et al*., 2007). Similarly, the neuroprotective actions of astrocytes decrease in PD (Mirza *et al*., 2000; Song *et al*., 2009), which shows a low level of glutathione in the SN (≈40%) (Sian *et al*., 1994) and an increased vulnerability of DAn to free radicals (Pearce *et al*., 1997). These facts are probably important for the onset and progression of PD (Halliday & Stevens, 2011).

On the other hand, astrocytes can actively induce neurodegeneration by different mechanisms, including the release of cytokines/TNFα (Lee *et al*., 2010) (e.g. in response to an excessive accumulation of α-synuclein) (Wakabayashi *et al*., 2000; Lee *et al*., 2010) and the release of glutamate (Morales & Rodriguez, 2012; Morales *et al*., 2013a,b). The astrocytic release of toxic agents can be facilitated by reactive astrogliosis, a global reaction of astrocytes to brain damage (Sofroniew & Vinters, 2010). Reactive gliosis has also been implicated in the deterioration of both the aging (Goss *et al*., 1991; Kohama *et al*., 1995; Hayakawa *et al*., 2007; Lynch *et al*., 2010) and the PD brain (Rogers *et al*., 2007; Werner *et al*., 2008). Thus, it is possible that an imbalance between the protecting vs. damaging actions of astrocytes on neurons during aging could also explain the marked action of aging on PD (Damier *et al*., 1993; Lin *et al*., 1993; Venkateshappa *et al*., 2012). The astrocyte population is normally replenished by new astrocytes derived from stem cells of the subventricular zone (Gonzalez-Perez & Quinones-Hinojosa, 2012; Mack & Wolburg, 2013). The reduction in gliogenesis induced by the senescence of the subventricular zone during the last third of life (Hoglinger *et al*., 2004; Galvan & Jin, 2007; Conover & Shook, 2011; Shook *et al*., 2012) is another circumstance to explain the aging of astrocytes and, consequently, the aging and death of DA neurons.

Microglia is normally found in a quiescent resting state with a small soma and highly ramified processes that contact neuronal synapses (Davalos *et al*., 2005; Nimmerjahn *et al*., 2005; Wake *et al*., 2009). In response to brain injury, the microglial cell shortens its branches, enlarges its somata, and expresses macrophage markers (Streit *et al*., 1989; Ito *et al*., 1998), thereby promoting the release of cytokines, chemokines, and ROS (Kreutzberg, 1996), and activating its migration (Kreutzberg, 1996) and phagocytic action (Streit, 2002; Doorn *et al*., 2012). This microglial reaction has been observed in PD (Hunot *et al*., 1996; Knott *et al*., 2000; Croisier *et al*., 2005; Orr *et al*., 2005), where it can be activated in response to aggregated (Zhang *et al*., 2005) and nitrated (Mor *et al*., 2003) forms of α-synuclein, thereby promoting the internalization and degradation of this protein (Zhang *et al*., 2005). Although the microglial reaction could be initially useful (e.g. preventing the accumulation of the α-synuclein in the Lewy bodies of DAn), its chronic activity can promote the DAn degeneration in PD (Halliday & Stevens, 2011). Similar chronic activation of microglia has been observed in the aging brain (Godbout & Johnson, 2004; Flanary, 2005; Gelinas & McLaurin, 2005; Streit *et al*., 2008; Campuzano *et al*., 2009) where these cells release high amounts of IL-6 and TNFα (Njie *et al*., 2012). In addition, it has been reported that senescent microglia does not perform its actions properly (e.g. it presents a shortening of the telomere), a fact that has also been suggested for the PD brain (Barcia *et al*., 2004; Ouchi *et al*., 2005; Mount *et al*., 2007; Marinova-Mutafchieva *et al*., 2009; Cunningham, 2013). Thus, the behavior of microglia also seems to be similar in the PD and aged brain.

Therefore, the action of aging on astrocytes and microglia seems to be important for the DAn degeneration observed in aging and PD. A similar conclusion is suggested by studies with pluripotent stem cells (Takahashi & Yamanaka, 2006) which *in vitro* differentiated to DAn. These neurons only present the typical characteristics of the degenerating parkinsonian DAn (reduced numbers of neurites, accumulation of a-synuclein, etc.) when they are aged in vitro (Sanchez-Danes *et al*., 2012; Isobe *et al*., 2014).

## The cause of PD and aging: the multifactor hypothesis

Although definitive conclusions about the etiology of PD have not been reached, it is generally considered that this illness is the consequence of the simultaneous action of a number of toxic and genetic agents able to degenerate the DAn in animals and humans (*multifactor hypothesis of PD*) (Olanow & Tatton, 1999; Bossy-Wetzel *et al*., 2004; Litvan *et al*., 2007a,b; Obeso *et al*., 2010). The combination of these damaging agents might not be exactly the same in all patients, which may explain the high clinical diversity observed in PD. A similar multifactor etiopathogenic hypothesis has been proposed for the brain aging of healthy subjects (Olson, 1987; Peto & Doll, 1997). As will be shown, most (if not all) agents included in the *multifactor hypothesis of PD* have also been included in the *multifactor hypothesis of aging*, thereby linking their etiopathologies (Table1) (see at the end of the section ?The cause of PD and aging: the multifactor hypothesis).

**Table 1:** Cell characteristics and mechanisms that are common to aging and Parkinson's disease (PD)

	Cellular changes				Etiology		
		PD	Age			PD	Age
SNn loss	Melanin+ cells	↓	↓	Pathogeny	Multifactorial	YES	YES
	TH+ cells	↓	↓	Oxidative stress	ETC disruption	YES	YES
	DD+ cells	↓	↓		ROS/RNS	↑	↑
	DAT+ cells	↓	↓		SOD activity	↓	↓
	Lat/Postdistribution	YES	YES		GPx activity	↓	↓
nsDAn adaptation	DA turnover	↑	↑		Lipid peroxidation	↑	↑
	DA receptors	↑	↑		Protein damage	↑	↑
	DAT activity	↓	↓	Mitochondrial dysfunction	mtDNA mutations	↑	↑
	DAn differentiation	↓	↓		mtDNA delections	↑	↑
PPN	Ach cells	↓	↓		Mitophagy	↓	↓
Locus coeruleus	NA cells	↓	↓		Transport	↓	↓
Thalamus	GLU cells	↓	↓	Genes/Proteins	Polygenic low penetrance	YES	YES
Astrocytes	Astrogliosis	YES	?		α-synuclein aggregation	YES	YES
	Glutation release	↓	?		Parkin activity	↓	↓
	Trophic factor release	↓	↓		UCH-L1 activity	↓	↓
	Cytokines, TNFα release	↑	↑		PINK1 activity	↓	↓
	GLU release	↑	?		DJ-1 activity	↓	↓
	Gliogenesis	↓	↓	Silent toxics	MPTP/paraquat …	↓	?
Microglia	μgliosis	YES	YES	Premotor disturbances	Olfactory dysfunctions	YES	YES
	IL-6/TNFα release	↑	↑		Sleep fragmentation	YES	YES
Stem cells	SVZ proliferation	↓	↓		Constipation	YES	YES
					Mood disorders	YES	YES

SNn, substantia nigra neurons; nsDAn, nigrostriatal dopamine neurons; TH+, cells with tyrosine hydroxylase immunoreactivity; DD+, cells with l-dopa decarboxylase immunoreactivity; DAT+, cells with immunoreactivity for the dopamine transporter; DA, dopamine; Ach, acetylcholine; NA, norepinephrine; GLU, glutamate; SVZ, subventricular zone; ETC, electron transport chain; ROS, reactive oxygen species; RNS, reactive nitrogen species; SOD, superoxide dismutase; GPX, glutathione peroxidase; mtDNA, mitochondrial DNA. Not all data summarized here have the same experimental or clinical confidence level. The question mark is included when available data are scarce, incomplete, or contradictory.

## Oxidative stress in PD and aging

The oxidative stress theory of aging proposed by Denham Harman in the 1950s states that the age-related loss of physiological functions is due to the progressive accumulation of oxidative damage (Harman, 1956). Nowadays, practically all theories implicate oxidative stress in cell aging (Gerschman *et al*., 1954; Brack *et al*., 2000; Toussaint *et al*., 2000; Jang & Van Remmen, 2009). The mitochondrial production of energy generates unpaired electrons (mainly in the complexes I and III) which facilitate the production of reactive oxygen (ROS) and nitrogen (RNS) species, damaging essential constituents of the cell (proteins, lipids, nucleic acids, etc.) (Sohal & Weindruch, 1996; Cakatay *et al*., 2003; Kayali *et al*., 2009; Murphy, 2009; Perez *et al*., 2009; Oliveira *et al*., 2010) and accelerating aging (Sohal & Brunk, 1992; Barja, 2002a). DAn could be particularly sensitive to oxidative stress and aging. The nsDAn has a very high number of synaptic terminals (up to 1 million per cell in humans) (Matsuda *et al*., 2009) and a thin unmyelinated axon (Orimo *et al*., 2011) which consume a high quantity of energy and which need local mitochondria to support their activity. If there were 10 mitochondria per synaptic terminals, then the total number of mitochondria in each DAn could be higher than 10 million, even when the axonal mitochondria are not included in the calculation. Bearing in mind that 0.2–2% of total oxygen consumption is converted into free radicals in the mitochondria (rather than into water) (Richter, 1992), the extremely high number of DAn mitochondria must generate a vast quantity of ROS whose toxic action during aging may be difficult to prevent. In addition, the DAn also produces ROS/RNS by other mechanisms, including DA metabolization by monoamine oxidase, DA autooxidation, and the Fenton reaction (these cells have a high concentration of iron) (Halliwell, 1992; Kidd, 2000; Ahlskog, 2005; Berg & Hochstrasser, 2006). Therefore, oxidative stress that promotes aging in all cells could be particularly dangerous in the DAn (Parker *et al*., 1989; Bender *et al*., 2006). The DAn is protected from free radicals by both nonselective mechanisms normally at work in all cells [superoxide dismutase (SOD) and glutathione peroxidase (GPX)] and selective mechanisms that are only operative in this cell. Examples of selective protecting mechanisms are the dopamine transporter (DAT) that moves DA from the extracellular to the intracellular medium, and the vesicular monoamine transporter 2 (VMAT2) that moves DA from the intracellular medium to synaptic vesicles. These agents protect DAn from the self-oxidation of DA, keeping the neurotransmitter away from the cell regions more vulnerable to oxidative stress and in an environment where the low pH decreases the oxidation rate.

Oxidative stress has been included in most models to explain the DAn degeneration in PD (Olanow & Tatton, 1999; Dawson & Dawson, 2003; Moore *et al*., 2005; Hwang, 2013). There is evidence showing a disruption of the mitochondrial electron transport chain (particularly of the complex I) which increases ROS generation in the PD brain (Parker *et al*., 1989; Shapira *et al*., 2002; Bender *et al*., 2006). This deficiency was also observed in platelets (Parker *et al*., 1989) and other tissues (Orth & Schapira, 2002) of patients with PD and was confirmed with cybrid models made with platelets of patients with PD (Swerdlow *et al*., 1996; Gu *et al*., 1998). The toxic effect of oxidative stress in the DAn has been tested in animals by administering drugs which, after blocking the mitochondrial complex I, induce a parkinsonian syndrome. These drugs include MPTP (Burns *et al*., 1983; Langston & Ballard, 1984; Ballard *et al*., 1985), rotenone (Ramsay *et al*., 1991; Betarbet *et al*., 2000), paraquat (Miller *et al*., 1978; Manning-Bog *et al*., 2002), and 6-hydroxydopamine (Ungerstedt, 1971; Rodriguez *et al*., 2001), which are drugs whose toxic action on the DAn have been contrasted in humans who received them unintentionally and who developed PD (Tanner, 1989; Semchuk *et al*., 1992; Gorell *et al*., 1998; Di Monte *et al*., 2002; Weisskopf *et al*., 2010). In addition, the mechanisms for nonselective (SOD and GPX; Riederer *et al*., 1989; Liu *et al*., 1994; Asanuma *et al*., 1998; Gonzalez-Zulueta *et al*., 1998) and selective (DAT and VMAT2) (Cruz-Muros *et al*., 2007a,b^2008^) DAn protection are downregulated in PD. The imbalance between the production of free radicals and the prevention of their toxic activities is probably at the basis of the high oxidative damage of lipids (Bosco *et al*., 2006), proteins, and DNA (Nakabeppu *et al*., 2007) found in the SN of the PD brain (Jenner, 2003, 2007). The same imbalance has been observed in the healthy aging brain (Sohal & Brunk, 1992; Sohal & Weindruch, 1996; Barja, 2002b,a; Cakatay *et al*., 2003; Kayali *et al*., 2009; Perez *et al*., 2009; Oliveira *et al*., 2010). In keeping with this, procedures that retard aging in animals (particularly caloric restriction) normally increase resistance to oxidative stress (Sohal & Weindruch, 1996; Yu, 1996; Yu & Yang, 1996; Barja, 2002b,a; Bokov *et al*., 2004). Thus, available evidence suggests a direct link between the age-related oxidative stress and the DAn degeneration in PD. Although oxidative stress is normally included in the etiopathology of PD (Subramaniam & Chesselet, 2013), there is no agreement about its origin. Oxidative stress could be induced by the action of other cells on the DAn (e.g. by excitotoxicity) (Nguyen *et al*., 2011) or may simply be the consequence of aging.

## Mitochondrial damage in PD and aging

Shortly after the discovery of the mitochondrial genome (mtDNA) and after the incorporation the mitochondrial damage to the oxidative stress hypothesis (Harman, 1972), the stress-related accumulation of mtDNA damage began to be considered as a major cause of aging (Miquel *et al*., 1980). Complex I is in close proximity to the mtDNA, and the inverse relationship between lifespan and ROS production (at complex I (Barja & Herrero, 2000; Lambert *et al*., 2007) has also been observed between lifespan and the number of mtDNA mutations (Vermulst *et al*., 2007). Human mtDNA is highly vulnerable to postnatal mutations not only because of its proximity to the electron transport chain (the source of 90% of ROS), but also because of its lack of protecting histones, and its high replication rate. If ROS-induced mtDNA mutations are not repaired quickly, then they will be propagated and fixed with the mitochondrial replication cycle where the G is replaced by T and C by A (Wang *et al*., 1998a). Thus, the mitochondrial replication produces a clonal expansion and a propagation of the mtDNA errors, amplifying tissue-specific mutations and inducing somatic mosaicism which increase with age (genetic drift) (Muller-Hocker, 1989, 1990; Fayet *et al*., 2002). The accumulation of mutations during life is much higher in the mtDNA (Chinnery *et al*., 1999) than in the nuclear DNA (Cantuti-Castelvetri *et al*., 2005; Reddy & Beal, 2005). As suggested by the 2'deoxyguanosine data, the mutating action of free radicals on aging is mainly induced in the mitochondrial genome, with the accumulation rate of the nuclear DNA mutations not being correlated with the maximum lifespan (Barja & Herrero, 2000). Sporadic random mutations of the mtDNA may not be enough to induce functional disturbances in young people. However, their accumulation throughout life deteriorates the normal physiology of cells (Cantuti-Castelvetri *et al*., 2005; Smigrodzki & Khan, 2005; Maruszak *et al*., 2006) and promotes aging (Harman, 1972; Linnane *et al*., 1989). Brain tissue is particularly susceptible to mutations (Vermulst *et al*., 2007), mostly when compared with other tissues which, as occurs with the liver, do not accumulate mtDNA mutations during most of a person's life (Ameur *et al*., 2011). The accumulation of the mtDNA deterioration with aging could be particularly high in the SNn (Reeve *et al*., 2013), which normally presents a high number of delections (>40% in healthy sixty-year-old subjects) (Bender *et al*., 2006). The influence of mtDNA on aging could help to explain why ultra-centenaries have a high content of mtDNA (He *et al*., 2014) and why the mtDNA background of some ethnic groups affects their lifespan (Raule *et al*., 2013).

The decrease in energy production is one of the consequences of mitochondria damage. The preservation of mitochondrial activity needs a number of mechanisms that consume a portion of the energy produced by the organelle. These energy-demanding mechanisms include those involved in the movement of mitochondria to the cell locus with the highest energy requirement (axons and synapses), in the repair of damaged mitochondria (including mtDNA), in the destruction of badly damaged mitochondria, and in the synthesis of new mitochondria. The healthy mitochondria produce much more energy than that required to maintain normal activity, a fact that changes with aging. The decrease in the energy resources in the aged cells may prevent the repair of the mitochondrial damage, thus precipitating the organelle in a vicious cycle (↓ energy production ↔ ↓ mitochondrial repair) where the mitochondria behaves as both cause and consequence of aging (Lauri *et al*., 2014). Mitochondrial damage similar to that found in the aging brain has been found in the PD brain where the accumulation of mtDNA mutations is usually higher in patients over 65 years of age (Kraytsberg *et al*., 2006). mtDNA delections in PD are a little higher than those observed in healthy aged subjects (52% vs. 44%) (Bender *et al*., 2006), data that also suggest that the difference between aging and PD is a matter of quantity more than a matter of quality.

Most data supporting the free radical theory of aging are correlative and do not directly prove this theory. On the other hand, there are data suggesting that the effect of oxidative stress on aging could be less relevant than frequently assumed. One way to directly test the free radical theory of aging is to manipulate the oxidative damage of mitochondria by modifying the expression of antioxidant proteins (Jang & Van Remmen, 2009). Studies with MnSOD, catalase, and thioredoxin knockout mice have provided inconclusive data to support the oxidative stress theory of aging (Muller *et al*., 2007; Jang & Van Remmen, 2009; Sohal & Orr, 2012). However, these knockout mice models of aging and other aging models such as the SAMP8 (Takeda, 2009; Shimada & Hasegawa-Ishii, 2011) or the mtDNA mutator mouse (Edgar & Trifunovic, 2009; Lauri *et al*., 2014b) could be particularly useful to study the influence of aging on the DAn degeneration in PD (Liu *et al*., 2008; Dai *et al*., 2013). In addition, different approaches have reported non-oxidative mitochondrial dysfunctions. A number of age-associated structural changes (e.g. enlargement of mitochondria, alterations of the cristae structure, matrix vacuolization/densification) have been reported (Wilson & Franks, 1975; Herbener, 1976; Tate & Herbener, 1976). Dysfunctional mitochondria may be repaired by a fission/fusion process. However, the mitochondria must be eliminated by mitophagy when the damage is important, a selective type of autophagy that maintains a suitable number of functional mitochondria and prevents the accumulation of those that are nonfunctional. Excessive mitochondrial stress (Ethell & Fei, 2009) or modifications of proteins involved in the mitophagy modulation (e.g. parkin and PINK1) (Narendra *et al*., 2010; Van Humbeeck *et al*., 2011; Palikaras & Tavernarakis, 2012) disturb mitophagy and promote PD. The low clearance of dysfunctional mitochondria involved in the progression of PD has also been linked to aging (Palikaras & Tavernarakis, 2012), which presents low autophagic activity (Cuervo *et al*., 2004; Hubbard *et al*., 2012) whose recovery (e.g. using caloric restriction) promotes longevity (Yen & Klionsky, 2008). Other autophagic processes whose modification have been linked to aging are probably also involved in PD (Cuervo *et al*., 2004; Martinez-Vicente *et al*., 2008; Wong & Cuervo, 2010; Hubbard *et al*., 2012). The cytosolic components of the mammalian cell may be degradated by three complementary processes: macroautophagy, microautophagy, and chaperone-mediated autophagy. These processes sequester proteins and organelles from the cytoplasm and deliver them into the lysosomal compartment where they are degraded and eliminated (Mizushima *et al*., 2008; He & Klionsky, 2009). The downregulation of these autophagic mechanisms has been involved in age-related neurodegeneration (Shibata *et al*., 2006; Palikaras & Tavernarakis, 2012). There is also increasing evidence showing insufficient autophagy in neurodegenerative disorders where this could explain the accumulation of proteins in cytosolic aggregates (e.g. of α-synuclein in Lewy bodies of PD) (Stefanis *et al*., 2001; Webb *et al*., 2003; Cuervo *et al*., 2004; Martinez-Vicente *et al*., 2008; Wong & Cuervo, 2010; Friedman *et al*., 2012). The identification of the autophagic disturbances in PD could provide new evidence to understand the age influence on PD, and perhaps for controlling both processes (Table1).

An efficient mitochondrial quality control needs mechanisms to move this organelle between different cell regions. The axon of the snDAn accounts for a high percentage of the cell volume (>95%) (Devor, 1999; De Vos *et al*., 2008) and uses a significant portion of its energy (Brookes *et al*., 2004). Thus, it contains a high number of mitochondria, a portion of which could be generated (Amiri & Hollenbeck, 2008) and restored (fusion/fission) (Court & Coleman, 2012) locally. However, only 13 of ≈1000 mitochondrial proteins are encoded by the mtDNA (Boore, 1999), which means that most synaptic and axonal mitochondria are probably synthesized in the neuronal somata and moved along the axon (>30% of axonal mitochondria are normally in motion) until reaching these distal structures (anterograde motion). In addition, axonal transport also moves dysfunctional mitochondria from synaptic bottoms and axons to the cell somata (retrograde motion) where they can be destroyed by mitophagy, lysosomes, and the ubiquitin-proteasome system (Cheng *et al*., 2010). Thus, the snDAn survival needs efficient axonal transportation (Sasaki *et al*., 2005; De Vos *et al*., 2008; Florenzano, 2012) which may be hampered by the small diameter (<1 μm) and the intensive branching (hundred thousands of bifurcations) of this cell axon (Matsuda *et al*., 2009). This transportation is probably damaged in PD. There is evidence suggesting that PD starts with axonal damage and progresses by a ‘dying back’ process to the DAn somata (Cheng *et al*., 2010). Proteins that collaborate with the kinesins/dyneins system to transport mitochondria across axons (e.g. α-synuclein, parkin and PINK1-Miro-Milton complex) have also been implicated in PD (Bueler, 2009; Weihofen *et al*., 2009; Yang *et al*., 2010). Axonal disruptions have also been observed in aged neurons (Fjell & Walhovd, 2010), which show a deterioration of axonal transport (Gilley *et al*., 2012) and of the quality control (Sterky *et al*., 2011) of the mitochondria. Thus, PD and aging also present similar anomalies in the axonal transport and quality control of mitochondria.

Other biochemical pathways that could be useful to compare the mitochondrial deterioration in aging and PD are beginning to be studied. Pathways involved in the modulation of microRNAs (miRNAs) are an example of these new possibilities. miRNAs are small noncoding RNA species that play crucial regulatory roles in many biological processes including those involved in the mitochondrial-mediated aging (Lauri *et al*., 2014). miRNAs can control senescence at multiple levels including the modulation of mitochondrial respiration, the modulation of genes involved in ATP and ROS production, and the regulation of autophagy (Zhao *et al*., 2010; Bai *et al*., 2011; Bandiera *et al*., 2011; Faraonio *et al*., 2012; Olivieri *et al*., 2012; Magenta *et al*., 2013). The modulating action of aging on miRNAs is probably different in each organ (e.g. in the liver and the brain), which could be helped to explain the different aging rate of tissues (Li *et al*., 2011). miRNAs are probably important not only for the ontogenic differentiation of the DAn (Junn & Mouradian, 2012), but also for its deterioration with aging and PD (Kim *et al*., 2007; Harraz *et al*., 2011; Mouradian, 2012). Perhaps after certain methodological improvements (e.g. after preventing the low stability of miRNAs in the brain samples), the comparison of the aging and PD miRNA could be particularly useful to identify similarities and differences between the DAn in both conditions (Sethi & Lukiw, 2009).

In summary, mitochondrial damage is a central fact in the aged and PD brain. Both aging and PD present a slow-progression mitochondrial disturbance linked to a slow-course neurodegeneration lasting for many years. The mitochondrial deterioration rate is not the same in all tissues (which could explain the different aging rates observed in the different organs of the body), with the DAn being particularly vulnerable to the aged mitochondria (Conley *et al*., 2007; Lauri *et al*., 2014). It is possible that some mitochondrial damages can be transmitted to the offspring, which could explain why the risk of developing PD may be greater in persons whose mother was affected by the disease than in those with affected fathers (Beal, 1998; Bonilla *et al*., 1999; Chinnery *et al*., 1999).

## Genetic control of PD and aging

There are data suggesting that aging is genetically programmed in animals and humans (Austad, 2004; Longo *et al*., 2005; Prinzinger, 2005). Humans present a significant degree of heritability of longevity, and a mortality rate doubling time (time to double the probability of dying) which is constant among human populations (≈8 years), even under different environmental conditions (Hjelmborg *et al*., 2006). However, the high number of genes involved (see GenAge database) and the partial relevance of most of them mean that genetic information cannot be used at present to predict the aging rate of individuals. This circumstance is also true for PD, where the polygenic low-penetrance and the gene–environment interaction has led to much debate about the actual significance of genetic factors on sporadic PD (Sellbach *et al*., 2006; Wirdefeldt *et al*., 2011). The offspring of long-lived parents seem to be protected against age-related diseases (Atzmon *et al*., 2004), which suggests that the genetic control of aging is involved in neurodegenerative illnesses. At present, there is not enough information to compare the polygenic control of aging and PD, but there is clear evidence showing that genes that modulate the DAn vulnerability (whose mutation induces familiar parkinsonisms) are linked to normal aging. Some of these genes will be briefly referred to below.

In general, genes that modulate the oxidative stress (Muftuoglu *et al*., 2004; Palacino *et al*., 2004; Devi *et al*., 2008; Irrcher *et al*., 2010) and the quality control of mitochondria (Bueler, 2009; Weihofen *et al*., 2009; Yang *et al*., 2010; Narendra *et al*., 2010; Van Humbeeck *et al*., 2011; Palikaras & Tavernarakis, 2012) have proved to be relevant for both PD and aging. α-synuclein (a protein involved in the recycling vesicles in DAergic synapses) (Abeliovich *et al*., 2000) may present anomalous conformations that facilitate the production of oligomeric species and amyloidogenic filaments whose aggregation and precipitation form the Lewy bodies found in nsDAn of the PD brain (Lansbury & Brice, 2002). There are data showing that oxidative stress can promote the aggregation of α-synuclein in PD and other synucleinopathies (Giasson *et al*., 2000). α-synuclein presents a similar behavior in aging, promoting the accumulation of pathogenic proteins, the impairment of the ubiquitin-proteasome system, and the loss of DAn (Li *et al*., 2004; Moore *et al*., 2005). In the PD brain, the anomalous activity of parkin (a protein involved in the ubiquitination of proteins and in mitophagy) produces an improper targeting of substrates for proteosomal degradation and in a fragmentation of the mitochondrial network (Lucking *et al*., 1998, 2000; Moore *et al*., 2005; Reeve *et al*., 2014). Similar facts have been reported for parkin during aging (Rodriguez-Navarro *et al*., 2007; Vincow *et al*., 2013). Changes in the UCH-L1 (a protein that recycles free ubiquitin) activity may result in an impairment of the efficiency of the ubiquitin-proteasome system (Osaka *et al*., 2003; Li *et al*., 2004) which is involved in PD (Leroy *et al*., 1998). This protein has been proposed as an intracellular stress marker in cell aging (Marzban *et al*., 2002). PINK1 phosphorylates mitochondrial proteins in response to cellular stress, thereby preventing mitochondrial dysfunctions and promoting (in cooperation with parkin) the degradation of damaged mitochondria by lysosomes and proteasomes (Valente *et al*., 2004a; Liu, 2014). The PINK1 activity is also involved in both PD (Valente *et al*., 2004b; Albanese *et al*., 2005; Gelmetti *et al*., 2008) and aging (Wood-Kaczmar *et al*., 2008; Vincow *et al*., 2013). DJ-1 is a ubiquitous protein located in neurons and glia, where it provides protection against oxidative stressors. The insoluble forms of DJ-1 are notably greater in the brains of patients with PD (Moore *et al*., 2005), and its mutation causes a familiar early onset of parkinsonism (Bonifati *et al*., 2003a,b; Ibanez *et al*., 2003). This protein has also been linked to aging (Marzban *et al*., 2002; Meulener *et al*., 2006).

Although the influence of the above-mentioned genes in different familiar parkinsonisms is clear, its actual relevance in sporadic PD is less evident. In addition to the above-mentioned genes, many other genes can influence lifespan. The possible influence of a gene on lifespan can be found in GenAge, a database with information on over 700 genes (Tacutu *et al*., 2012; Budovsky *et al*., 2013). The possible relevance of most of these age-related genes on PD cannot be directly checked because there is not a gene bank for PD similar to GenAge (Wirdefeldt *et al*., 2011). Thus, it is not possible to compare the configuration of genes involved in PD and aging at present. Another limiting fact to consider is the complex genome–environment interaction that modulates the aging process and that probably influences the risk of PD. The regulation of single genes can extend the lifespan of some primitive organisms more than 10-fold (Ayyadevara *et al*., 2008). Although the genetic modulation of lifespan is more complex and less efficient in mammals, the advance in knowledge about the interaction of the age-related genes and the environment is starting to be used to extend the lifespan of these animals (de Magalhaes *et al*., 2012). Thus, the aging of certain mammals may be modulated by manipulating external (e.g. temperature, diet) and internal (e.g. by targeting the insulin-like group factor receptors, growth hormone receptors, sirtuins) variables (de Magalhaes *et al*., 2012; de Cabo *et al*., 2014). If, as the present review suggests, the age-related mechanisms are directly involved in PD, then the control of some of the environmental variables that have proved useful to modulate aging in animals (e.g. caloric restriction, hormesis, spermidine) could also be useful to control the onset and progression of PD. Some of these variables have already proved their efficacy in animal models of PD. This is the case of the caloric restriction whose utility to control aging has been proved in practically all animal species studied and which may decrease the vulnerability of the DAn to toxic agents (Duan & Mattson, 1999). There is some epidemiological (de Lau *et al*., 2005) and clinical (Vanitallie *et al*., 2005; Nunomura *et al*., 2007) studies suggesting that the caloric restriction and the control of diets (antiglycating agents..) could decrease the clinical expression and the evolution of PD (Wirdefeldt *et al*., 2011; Hipkiss, 2014). Sirtuin 1, a protein that mediates longevity under calorie restriction (Jadiya *et al*., 2011; Zhang *et al*., 2011), decreases in DAn which received dopaminergic neurotoxics such as rotenone, 6-hydroxydopamine, or MPTP (Alvira *et al*., 2007; Pallas *et al*., 2008; Albani *et al*., 2009; Jadiya *et al*., 2011; Srivastava & Haigis, 2011). The overexpression of sirtuin 1 (Wareski *et al*., 2009) or its activation by resveratrol (Okawara *et al*., 2007; Chao *et al*., 2008; Jin *et al*., 2008) decreases the action of these toxics on the DAn. This protective effect has not been shown for other sirtuins (Dillin & Kelly, 2007) which, as occurs with the sirtuin 2, may even promote the cell death in animal models of PD (Outeiro *et al*., 2007). Other agents that could be useful for modulating both aging and PD are the insulin growth factors (Mattson *et al*., 2004; de Cabo *et al*., 2014; Numao *et al*., 2014), spermidine (Pucciarelli *et al*., 2012; De La Hera *et al*., 2013), metformin (Patil *et al*., 2014), and rapamycin (Malagelada *et al*., 2010; Liu *et al*., 2013).

## Aging, silent toxics, and PD

The incidence of PD increases sharply toward the end of the fifth decade of life just as the effect of aging all over the body appears. The DAn degeneration considered as the hallmark of PD has also been observed in the aged brain where it is induced by most (if not all) agents involved in PD. The main difference between DAn degeneration in PD and aging seems to be a question of quantity (degree of cell loss) more than a question of quality (presence/absence of cell loss; McGeer *et al*., 1977; Kordower *et al*., 2013). Senescence is a wider phenomenon affecting practically all cells in the body, whereas PD has been restricted to some brain centers and cell populations. The present review suggests that PD may be the local expression of aging in some cell populations whose characteristics (high number of synaptic terminals and mitochondria, unmyelinated axon) make them highly vulnerable to aging. If PD is the result of aging, a key question to consider is why only 4-5% of people develop the illness. In addition to the genetic characteristics of each individual, the environmental circumstances affecting each person throughout their life are different. Many circumstances capable of increasing DAn vulnerability present a toxic action which is not traumatic enough to induce clinical signs of PD in the short term (‘silent toxics’). Subjects who are highly exposed to silent toxics could present PD if their lifespan is long enough, whereas other people who are less exposed to silent toxics or with a short lifespan will never present PD. In these subjects, the compensatory mechanisms of the surviving DA neurons are sufficient to maintain the basal ganglia activity, and the age-related DAn loss induced by aging does not reach the threshold that triggers the motor disturbances. Thus, in these subjects (≈94% of people under 70), the motor disturbances are not evident and the DAn degeneration goes unnoticed.

There are many silent toxic candidates. This is the case of pesticides and other chemical agents whose frequent presence in the environment may explain why farming, well water use, and rural living (normally accompanied by occupational exposure to herbicides, insecticides, and other chemicals) increase the risk of PD (Seidler *et al*., 1996; Gorell *et al*., 1998; Di Monte *et al*., 2002; Coon *et al*., 2006; Hancock *et al*., 2008; Weisskopf *et al*., 2010). Other possible damaging agents could be the high consumption of dietary products or low physical activity (Wirdefeldt *et al*., 2011). Some silent toxics could even perform their damaging action in prenatal life. The exposure of pregnant animals to neuroactive agents can produce permanent effects in the monoaminergic neurons of the offspring (Alonso *et al*., 1991a,b; Arevalo *et al*., 1991). The pre- or neo-natal exposure of rats to gram(−) bacteriotoxin lipopolysaccharide (LPS) induces a permanent decrease in the number of DA neurons (Ling *et al*., 2002; Carvey *et al*., 2003; Fan *et al*., 2011a,b; Cai *et al*., 2012) and an increase in the α-synuclein aggregation in the surviving DAn (Bandiera *et al*., 2011). The identification of silent toxics is not easy because subjects are normally exposed to a myriad of chemical mixtures of environmental pollutants during their life and because the effect of these toxics may be modulated by the genetic ‘fingerprint’ of each subject (Paolini *et al*., 2004). Some of these silent toxics probably also increase the aging rate in subjects without PD. There is an increasing number of studies showing that some external agents that accelerate aging are similar to those that increase the risk of PD (aging epigenome) (Sinclair & Oberdoerffer, 2009). Thus, what the silent toxic may be doing is accelerating the effect of aging on the DAn, with PD always being a matter of time. This possibility could explain why PD went practically unnoticed until life expectancy increased the way it did over the last two centuries. In the same way, if life expectancy continues to increase in the future, and the mechanisms involved in aging are not controlled, an increase in the percentage of the population suffering PD is to be expected.

## PD and aging: a final overview

In summary, the present review suggests that PD is the result of the slow neurodegenerative action of aging, an effect that can be accelerated by repeated damage to DAn accumulated over a person's lifespan. When the DAn degeneration reaches a critical level and the compensatory mechanisms are insufficient to maintain the basic functions of dopamine, the first motor disturbances appear and the diagnosis of PD can be made. Thus, the etiologic agents involved in PD could be the same as those involved in aging. The action of these agents could be particularly important in DAn because of their high vulnerability to age-related agents and because these cells are highly susceptible to a number of silent toxics (Going *et al*., 2002; Takubo *et al*., 2002; Jeyapalan & Sedivy, 2008; de Cabo *et al*., 2014). This could explain why 50 years after first finding of DAn degeneration in sporadic PD, no specific causes for this illness have yet been found. In our opinion, more direct attention to the aging processes could accelerate the acquisition of new knowledge on the biological basis of PD, and actions aimed at delaying aging or promoting rejuvenation could also be useful to control the onset and progression of PD (Medvedev, 1981; Holliday, 1984). On the other hand, the considerable effort that is being made all over the world to understand the mechanisms involved in neurodegenerative illness should also contribute to clarifying the biological basis of aging.

## Funding

No funding information provided.

## Conflict of interest

None declared.
